# Redox-responsive self-assembly PEG nanoparticle enhanced triptolide for efficient antitumor treatment

**DOI:** 10.1038/s41598-018-29692-0

**Published:** 2018-08-28

**Authors:** Yanchun Wang, Xuewei Liu, Xuemei Wang, Wei Zheng, Junping Zhang, Feng Shi, Junbao Liu

**Affiliations:** 1 People’s Hospital of Zhengzhou University, Zhengzhou, 450003 Henan P.R. China; 2 Henan Provincial People’s Hospital, Zhengzhou, 450003 Henan P.R. China; 3 Henan University of Traditional Chinese Medicine, Zhengzhou, Henan P.R. China; 4 Henan Academy Institute of Traditional Chinese Medicine, 45000 Zhengzhou, P.R. China; 5 Jiangsu University, 212013 Zhenjiang, P.R. China

## Abstract

Chemotherapy induces tumor cell death by directly damaging DNA or hindering cell mitosis. Some of the drawbacks of most chemotherapy are lack of target selectivity to tumor cells, and adverse drug reaction, which limit the treatment intensity and frequency. Herein, we synthesized the prodrug of triptolide (TP) coupled to vitamin E (VE) using dithiodiglycolic acid and co-dissolved with PEG2000- linoleic acid (MPEG200-LD) in ethanol. The PEGylated TP prodrug self-assembly nanoparticles (PTPPSN) were prepared via nanoprecipitation method. Besides, characterization, stability and *in vitro* release of the PEGylated nanometer prodrug were investigated. Furthermore, *in vitro* and *in vivo* antitumor efficacy of PTPPSN explored showed that the cytotoxicity of triptolide was significantly reduced *in vitro* preparation. However, *in vitro* and *in vivo* antitumor effect of PTPPSN was significantly improved compared to the original triptolide. In summary, the PEGylated nanoparticle successfully encapsulated triptolide yielded suitable cell microenvironment, and nanotechnology-related achievements. This study, therefore, provides a new method for antitumor research as well as an innovative technology for clinical treatment of malignant tumor.

## Introduction

A malignant tumor is a grave threat to human life, and its morbidity and mortality are rapidly increasing on yearly basis. The utilization of small-molecule compounds as chemotherapeutic agents can directly damage DNA^1^ or hinder cell mitosis^2^, and induce cell death^3,4^. However, drawbacks such as lack of chemotherapeutic selectivity which causes toxicity to healthy cells^5^, alongside adverse drug reaction^6,7^, limit the treatment intensity and frequency of administration of such agents. Chemotherapy does not only damage the function of heart, liver, lung, kidney, bone marrow and other vital organs but also destroy the immune system^8^, resulting in the loss of the body’s self-protection barrier to the tumor^9^. Additionally, chemotherapy aggravates tumor cell genome instability^10^, which in turn causes the tumor cells to adapt to chemotherapeutic drugs rapidly^11,12^. Therefore, the side reactions of chemotherapy and tumor resistance have become major obstacles to the treatment of cancer patients^13,14^.

Triptolide (generally known as TP or TL), a specie of diterpene lactone epoxide compound, is extracted from traditional Chinese medicinal plant *Tripterygium wilfordii Hook. F*. Triptolide is considered as one of the main active ingredients in the plant^15^. Reports on treatment of multiple cancers using triptolide showed that cell proliferation inhibition and cell cycle arrest of the drug is dependent on time and doses^16–18^. During triptolide therapy, LC3-α expression level increases^19,20^, PI3K - Akt - mTOR pathway is inhibited^21,22^, while ERK1/2 is activated, but autophagy of cell death is inducted. Due to its double-edged effect on efficacy and toxicity, the treatment dose is closer to the toxic dose. Therefore, the safety of triptolide is of great health concern to the users. Based on in-depth investigation on the structure-activity relationship of triptolide, some efficient and less toxic derivatives of triptolide have been synthesized^23–26^. The structural modification of triptolide has partly resolved its high toxicity and poor solubility. However, clinical research of triptolide derivatives are stagnated in phase I trials, a development that has reduced subsequent research and reports on the drug. We, therefore, speculated that the high toxicity of triptolide alongside low selectivity to diseased cells, while normal cells could account for the retarded clinical study. Presently, the application of triptolide has not been improved, hence, additional research on the structural modification and targeted delivery of triptolide need to be entrenched.

The broad application of nanotechnology in the field of drug delivery has played an active role in designing and building new and efficient conveyance system, which has significantly enriched drug delivery strategies and the development of biomaterials. In the field of anticancer drug delivery, for instance, nano drug carrier has unique advantages such as improving the drug effect^27^; prolonging drug duration in blood circulation^28^; passive targeting to the tumor site via EPR effect^29^; improving uptake of drugs with active targeted modification by tumor cells^30^; as well as controlling drug release in the cellular targeted area^31^. Although, there has been great progress in nanotechnology delivery system, some setbacks including low efficiency, poor stability, toxic side effects of carrier materials, crystallization and leakage of drugs during storage, still hamper their clinical applications.

As compared with normal cells, the microenvironment of tumor cells is oxidatively stressed due to the excessive joint production of glutathione (GSH) and reactive oxygen (ROS) in tumor cells^32^. This particular ROS microenvironment has been widely used in the design of REDOX stimulation responsive drug delivery systems for anticancer drugs delivery. For example, disulfide bonds have been broadly explored in developing and restoring sensitive prodrug and nano drug delivery systems^33^. The disulfide bond of TP prodrug can be rapidly fracted to reveal the free mercapto under the action of GSH, while the acyl bond is rapidly hydrolyzed. Also, many studies have shown that tumor microenvironment exists with REDOX heterogeneity, namely different tumors may produce varied levels of glutathione and reactive oxygen species^34–36^. Furthermore, different regions of the same tumor might also possess the REDOX heterogeneity of cell microenvironment, and this could be observed in the diverse growth stages of the tumor.

**Figure 1:**
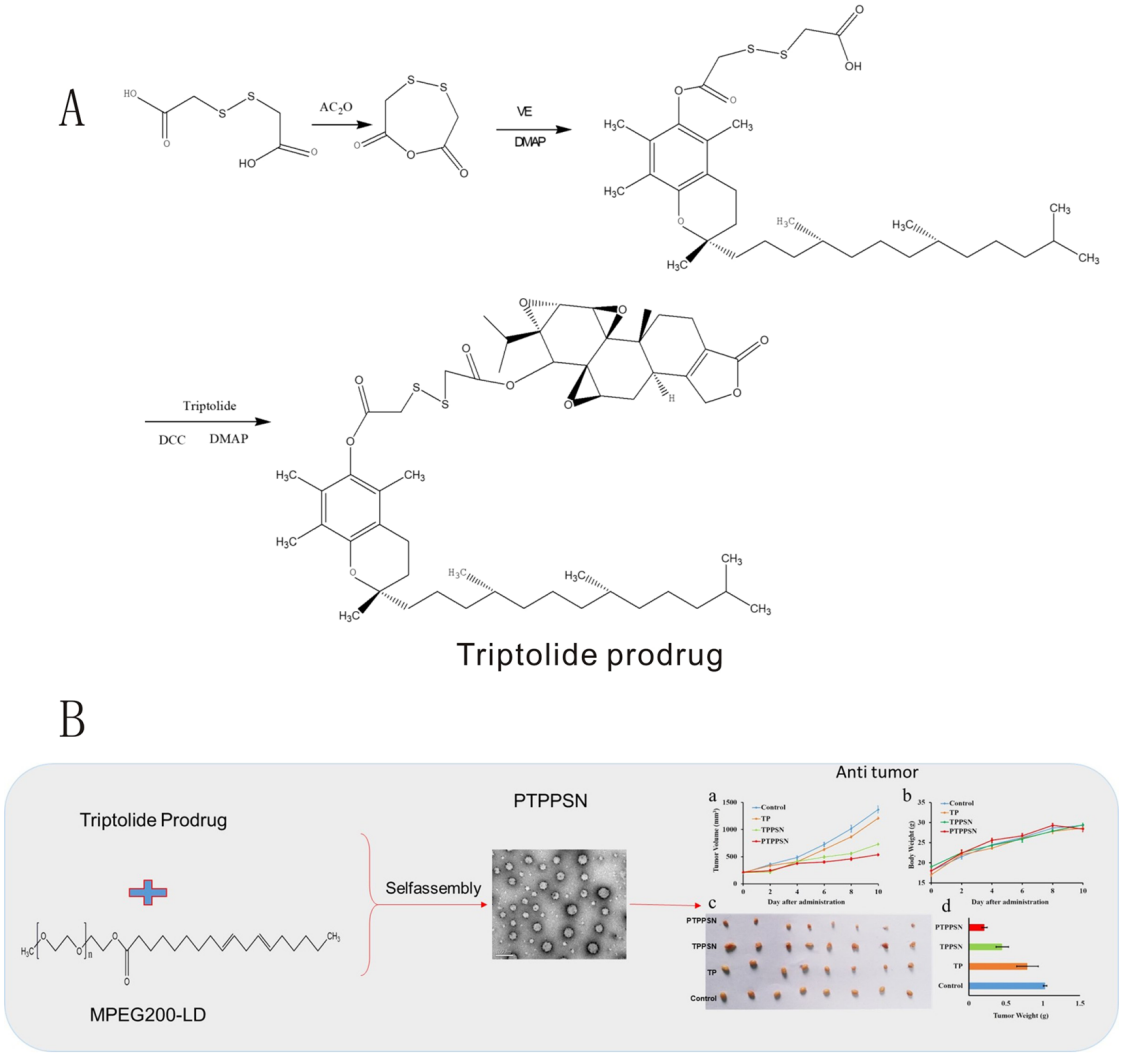
The Schematic diagram of triptolide prodrug (**A**) and redox-responsive self-assembly PEG nanoparticle for antitumor of triptolide (**B**). (a) antitumor effect in terms of tumor growth (error bars are mean ± SD, n = 10); (b) the change of body weight during the treatments; (c) tumor growth after systemic application of different treatment groups; (d) tumor weight (error bars are mean ± SD, n = 10).

In this paper, with the bifunctional chelating agent of dithiodiglycolic acid, a precursor drug was formed with vitamin E and triptolide (TP-S-S-VE), and co-dissolved with MPEG200-LD in alcohol. The PEGylated TP prodrug self-assembly nanoparticles (PTPPSN) were prepared via nanoprecipitation method. Besides, characterization of the nanometer prodrug, stability and *in vitro* release were also investigated. Furthermore, the antitumor efficacy of the nano prodrug was explored, and the results showed that the cytotoxicity of triptolide was significantly reduced *in vitro*, while the *in vitro* and *in vivo* antitumor effect of PTPPSN was significantly improved compared to the free triptolide. The sketch of this issue can be seen in Fig. 1.

## Methods and Materials

### Materials

The TPL and CL were purchased from Chengdu Biopurify Phytochemicals Ltd. (Chengdu, China), while the cocoons were kindly supplied by Tongxiang mulberry silk base of Zhejiang Province (Tongxiang, China). Dialysis membrane of 7,000 Da (MWCO) was obtained from Viskase Companies, Inc. (Chicago, USA). Hoechst 33342 and 3-[4, 5-dimethylthiazol-2-yl]-2, 5-diphenyltetrazolium bromide (MTT) were purchased from Sigma-Aldrich (St. Louis, MO, USA). RITC and Annexin V-FITC apoptosis detection kits were procured from Sigma Chemicals (St. Louis, MO, USA). HPLC grade acetonitrile and methanol were also obtained from BDH Chemicals (Gibbstown, NJ, USA). Ethanol and acetone were acquired from VWR international (Darmstadt, Germany) unless specified otherwise. Other chemicals used in this work were all of analytical pure grade and were used as received. Dulbecco’s modified Eagle’s medium (DMEM) and fetal bovine serum (FBS) were purchased from Gibco BRL (Carlsbad, CA, USA). Additionally, Penicillin-streptomycin, 0.25% trypsin-EDTA and non-essential amino acid were obtained from Invitrogen Co. (Carlsbad, CA, USA). Human pancreatic cancer cells MIA PaCa-2 and Panc-1 cells were generous gifts from Dr. Prabhu’s laboratory, originally obtained from American Type Culture Collection (Rockville, MD, USA).

### Synthesis, purification, and characterization of triptolide prodrug

All the experiments protocols were approved by the principles of laboratory animal care and legislation in force in Zhengzhou University. The synthesis of triptolide prodrug (0.2 g) was obtained by reacting the corresponding dithiodiglycolic acids with vitamin E (VE), followed by grafting of triptolide. Accordingly, the required amount of acid was added to acetic anhydride and stirred for 2 h at 30 °C. Afterward, toluene was added to the reactive system, prior to total removal of toluene and dithiodiglycolic acid with a rotary evaporator. The residual oily liquid was dissolved in 2 mL of anhydrous dichloromethane. Subsequently, 0.1 g of VE and 4- dimethyl aminopyridine (DMAP) were added and stirred at room temperature. The mixed solution was purified using silica gel column chromatography and subsequently washed with n-hexane/ethyl acetate/acetic acid (10/1/1) to obtain VE-S-SCOOH (114 mg). Triptolide (100 mg) and VE-S-SCOOH (120 mg) were dissolved in 6 mL anhydrous dichloromethane, and added to dicyclohexylcarbodiimide (DCC, 48 mg) alongside DMAP (30 mg), and was stirred for 2 h at room temperature. Afterwards, the solution was filtered to remove dicyclohexylurea (DCU) and further purified via silica gel column chromatography with an eluent of petroleum ether/ethyl acetate anhydride (2/3) to collect the TP prodrug (80 mg). The target product was characterized using ^1^H-NMR.

### Synthesis, purification, and characterization of mPEG2000-LD

Linoleic acid (500 mg), mPEG2000 (1000 mg), DCC (400 mg) and DMAP (240 mg) were dissolved in 20 mL anhydrous dichloromethane and stirred for 12 h. The solution was filtered to remove DCU, washed with saturated sodium chloride solution, and then dried over anhydrous sodium sulfate. The organic layer was filtered, rotary evaporated to dryness and washed thrice with petroleum ether. The insoluble substance was filtered and was characterized as mPEG2000-LD by ^1^H-NMR.

### PEGylating TP prodrug nanoparticle preparation

One-step nano sedimentation method was applied to prepare the nanoparticles^37^. Different ratios of mPEG2000-LD were added to a fixed portion of triptolide prodrug ethanol solution (20 mg/mL) (TP prodrug:mPEG2000-LD, 1:0.1; 1:0.2; 1:0.4 and 1:0.8) under a stirring condition. Each mixture was slowly dropped into double distilled water. The alcohol in the solution was rotary evaporated to obtain different formulations of PEGylated TP prodrug self-assembly nanoparticle (PTPPSN). TP prodrug self-assembly nanoparticle (TPPSN) was also prepared with the same process without mPEG2000-LD.

### The characterization of PTPPSN

The different proportions of self-assembled nanoparticles were characterized by particle size, zeta potential, polydispersity index, drug-loading capacity, and encapsulation rate indices. The DLS, TEM, and HPLC were adopted to investigate the effect of factors such as varying ratios of TP prodrug and mPEG2000-LD as well as temperature. Detailed parameters investigated included particle size and zeta potential detected by DLS (where the nanoparticles were diluted with deionized water to a concentration of 1 mg/ml prior determination at 25 °C using Zetasizer Nano ZS, Malvern Instruments); morphology observed with TEM; and the structure measured via ^1^H-NMR. The HPLC was performed at 25 °C using a C18 column (150 mm × 4.6 mm, 5 μm, Waters, USA) and methanol-water (42:58, v/v) as mobile phase which flowed at a rate of 1 mL/min. The detection wavelength was 218 nm. Encapsulation efficiency (EE) and drug loading (DL) of the nanoparticles were calculated according to equations (1) and (2):

1 $${\rm{DL}}( \% )=1-{\rm{Amount}}\,{\rm{of}}\,{\rm{the}}\,{\rm{drug}}\,{\rm{in}}\,\mathrm{NPs}/\mathrm{Amount}\,{\rm{of}}\,{\rm{the}}\,{\rm{NPs}}\times \mathrm{100} \% $$

2 $${\rm{EE}}( \% )={\rm{1}}-{\rm{Amount}}\,{\rm{of}}\,{\rm{the}}\,{\rm{freed}}\,\mathrm{drug}/\mathrm{Amount}\,{\rm{of}}\,{\rm{the}}\,{\rm{drug}}\times \mathrm{100} \% $$

### Stability experiment

Each formulation of PTPPSN as well as TPPSN was dispersed in the Phosphate Buffer Solution (PBS, pH = 7.4) under normal temperature. The particle sizes were measured at various time points viz., 1, 2, 3, 5, 10, 15, 20, 25, and 30 days to evaluate the stability of each formulation.

### Drug release

The cumulative release kinetics of the optimized PTPPSN, TPPSN and TP were determined in different concentration of GSH in PBS. A 100 μL of each preparation (1 mg/mL TP equivalent) was placed in 25 mL of the medium at 37 °C water bath. A portion of the solution (100 μL) was taken at different time points (0.5, 1, 2, 4, 8, 12, 24 h) and dissolved in 300 μL methanol. The TP concentration of samples were determined using HPLC, and the release rates were calculated.

### Cell proliferation assay

MTT [3-(4,5-dimethylthiazol-2-yl)-2,5-diphenyl tetrazolium bromide] was used to test cytotoxicity of TP prodrug self-assembly nanoparticle and cell viability. Briefly, 2 × 10^5^ of H_22_ BALB/C mouse liver cancer cell (H_22_) per well were incubated in 200 μL medium containing 10% FBS in 96-well plates for 24 h. The cells were then exposed to series of TP, TP prodrug, TPPSN, and PTPPSN for 72 h. After drug exposition, the medium was removed and 100 μL of MTT solution (0.5 mg/mL in DMEM containing 10% FBS) was added to each well. The plates were incubated for 2 h at 37 °C and 20 μL DMSO solution was then added to each well for 4 h at 37 °C. Absorbance was measured at 570 nm using a plate reader (Biotech America). The percentage of surviving cells was calculated as the absorbance ratio of treated to untreated cells. The inhibitory concentration 50% (IC50) of the treatments was determined from the dose-response curve. All experiments were set up in quadruplicate to determine means and SDs.

### *In vivo* antitumor study designs

All animals were cultured in SPF condition during the experimental period and handled according to the principles of laboratory animal care and legislation in force in Zhengzhou Univerisity. 18–22 g Kunming mice were purchased from Zhengzhou Univerisity. The 1 × 10^7^ of H_22_ cells were injected subcutaneously into mice toward the upper portion of the right flank, to develop a solid tumor model. Tumors were allowed to grow until a volume around 1 cm^3^ before initiating the treatment. Tumor length and width were measured with calipers, and the tumor volume was calculated using the following equation: Tumor volume (V) = 0.52 × length × width^2^. Tumor-bearing nude mice were randomly divided into 4 groups (n = 10) and each group, respectively, received intravenous injections through the lateral tail vein as follows: Group 1: PTPPSN at a triptolide equivalent dose of 7 mg/kg; Group 2: TPPSN at a triptolide equivalent dose of 7 mg/kg; Group 3: triptolide 7 mg/kg and Group 4: saline 0.9% as blank. The mice were regularly monitored for changes in tumor size and weight.

### Tissue distribution

The tumor model mice were grouped and injected intravenously through the lateral tail vein with either TP, TP prodrug, TPPSN, or PTPPSN. After intravenous injection, blood, heart, liver, spleen, lung, kidney and tumor were taken from the mice at different times (0.25, 0.5, 1, 2, 3, and 4 h). The blood sample was drawn into a 2 mL tube containing 10 μL of sodium heparin and was centrifuged at 3000 × g for 10 min to obtain the plasma, before storage at −20 °C pending further determination. Each organ was precisely taken and added to 0.1 g/mL normal saline. After crushing with high-speed shears, the samples were kept and stored in the tubes at −20 °C for HPLC detection.

A 150 μL of plasma or 300 μL of the organ homogenate was added to 10 μL internal standard, prednisolone (4 μg/mL) dissolved in 1 mL of methanol. All the mixtures were vortexed for 5 min and added to 2 ml of ethyl acetate, while vortexing for 10 min. The vortexed solution was centrifuged at 8000 × g for 10 min to obtain the organic layer. The upper organic layer was dried using nitrogen blown under 40 °C water bath. The dried samples were resolved with 100 μL of the mobile phase, and centrifuged at 8000 × g for 10 min to obtain supernatant for HPLC determination.

The HPLC was performed at 25 °C using a C18 column (150 mm × 4.6 mm, 5 μm, Waters, USA), while methanol-2 mM ammonium acetate (42:48, v/v) were used as mobile phases at a flow rate of 1 mL/min. The detective wavelength was 218 nm.

## Results

### Synthesis of TP produg and mPEG2000-LD

The bioconjugate, TP prodrug, was obtained through the reaction of dihydroxyacetic acid (Fig. 1A). The target product, TP prodrug was dried and tested using ^1^H-NMR (400 MHz, CDCl3, ppm): δ 4.98(2 H, s), 4.56(1 H, t), 4.17(1 H, t),3.88(4 H, m), 3.38(2 H, d), 2.45–2.72(12 H, m), 2.41(1 H, t), 1.96(5 H, m), 1.65(8 H, m), 1.31(16 H, m), 1.05(18 H, m). The results showed that TP prodrug was successfully synthesized by ^1^H-NMR.

**Figure 2:**
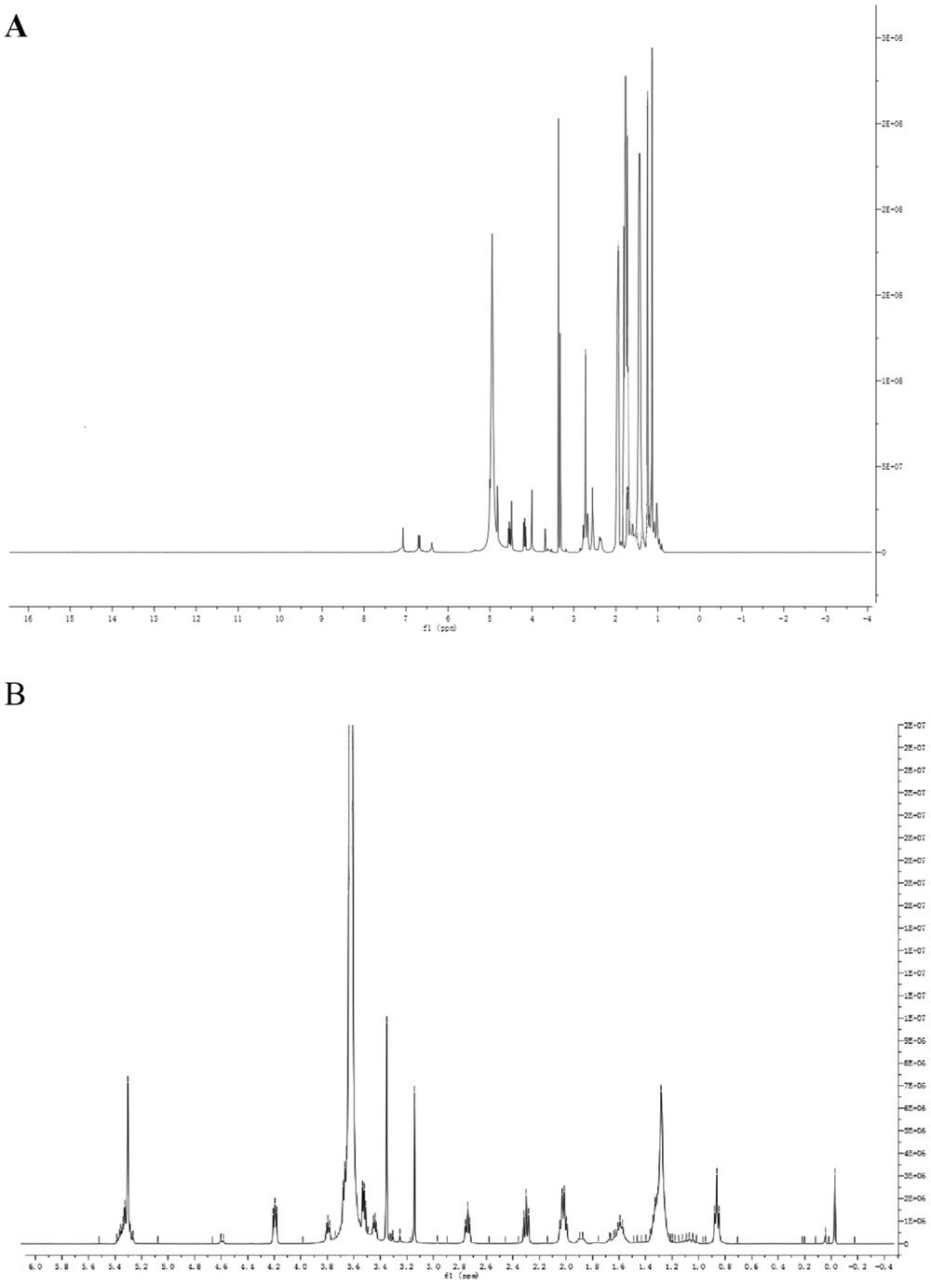
Identification of synthesized products. (**A**) ^1^H-NMR of TP PRODRUG; (**B**) ^1^H-NMR mPEG-LD.

The target product of mPEG2000-LD was dried and tested using ^1^H-NMR. ^1^H-NMR (400 MHz, CDCl3, ppm): δ 5.23 (6 H, m), 4.15 (2 H, t), 3.58 (180 H, s), 3.31 (3 H, s), 3.11(2 H, s), 2.69–2.72 (2 H, t), 2.25–2.32 (2 H, t), 1.84–1.96 (4 H, s), 1.18–1.38 (15 H, m), 0.83 (3 H, t). The result showed that mPEG2000-LD was successfully synthesized (Fig. 2B).

### Synthesis, morphological characterization and physicochemical properties of self-assembly nanoparticles

PTPPSN was prepared via different ratios of mPEG2000 nanoparticles, and the particle size and zeta potential were characterized by SEM and dynamic light scattering. The results as shown in Table 1 indicate that with an increase in mPEG2000-LD concentration, the particle size of the PTPPSN also decreased, with a steady rise in zeta potential.

**Table 1:** Physicochemical characterization of the PTPPSN (mean values ± standard deviation, n = 3): measurement of mean diameter (d), Zeta potential (z) and polydispersity index (PDI).

Ratio	Z(mV)	D(nm)	PDI
1:0.1	−28.47 ± 1.32	189.23 ± 2.68	0.078 ± 0.009
1:0.2	−26.64 ± 1.27	176.34 ± 2.53	0.085 ± 0.019
1:0.4	−23.18 ± 1.18	155.27 ± 2.15	0.064 ± 0.017
1:0.8	−21.74 ± 1.12	126.09 ± 1.68	0.067 ± 0.023

**Figure 3:**
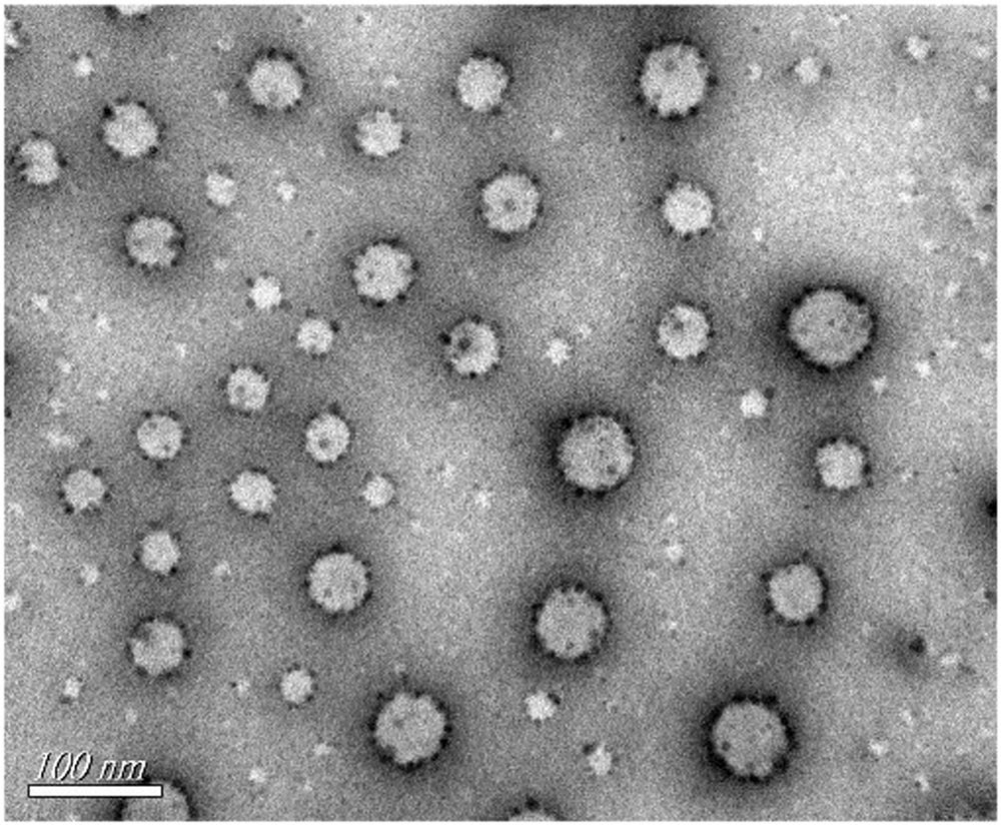
TEM image of PTPPSN.

As shown in the TEM images in Fig. 3, TP prodrug self-assembly nanoparticles were spherical in shape and monodispersed.

### Drug loading capacity and encapsulation efficiency

Drug loading capacity (DL) and encapsulation efficiency (EE) play critical roles in drug delivery. To obtain high drug loading to meet therapeutic needs, the optimized formulation was used to prepare TP prodrug self-assembly nanoparticle. The DL of the prepared PTPPSN was 57.0 ± 4.7%, along with an EE of 81.8 ± 2.8%.

### Stability test

**Figure 4:**
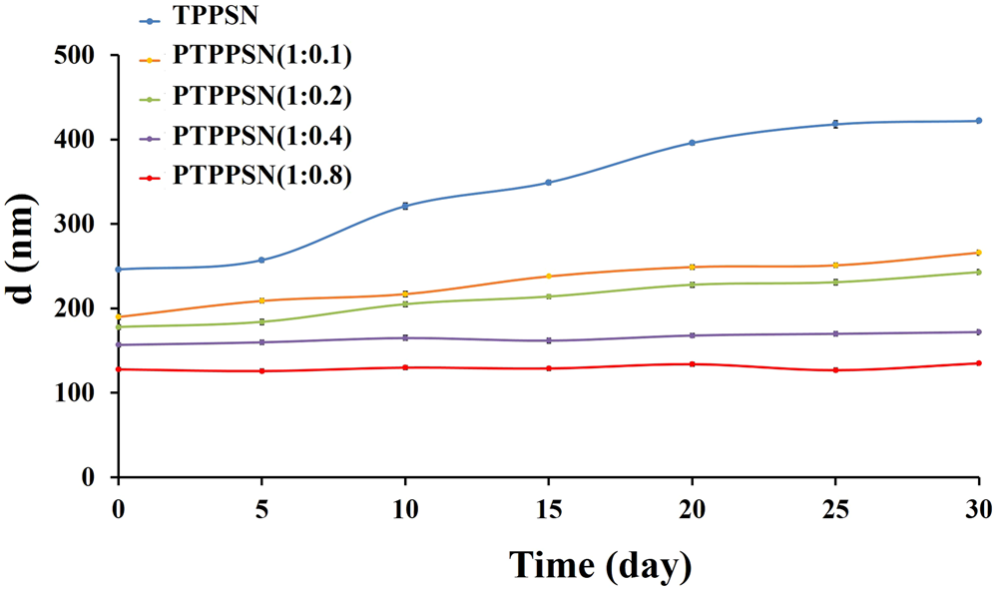
Stability of different formulations of PTPPSN in PBS (1:0, 1:0.1, 1:0.2, 1:0.4, 1:0.8). The values are represented as mean ± standard.

As shown in Fig. 4, the particle size of TPPSN increased gradually in the medium with the extension of time. However, when the mPEG - LD/TP - S - S - VE ratio was greater than 0.2, the particle size had no obvious change. The results showed the PEGylation can improve the stability of the nanoparticles.

### *In vitro* drug release profile

**Figure 5:**
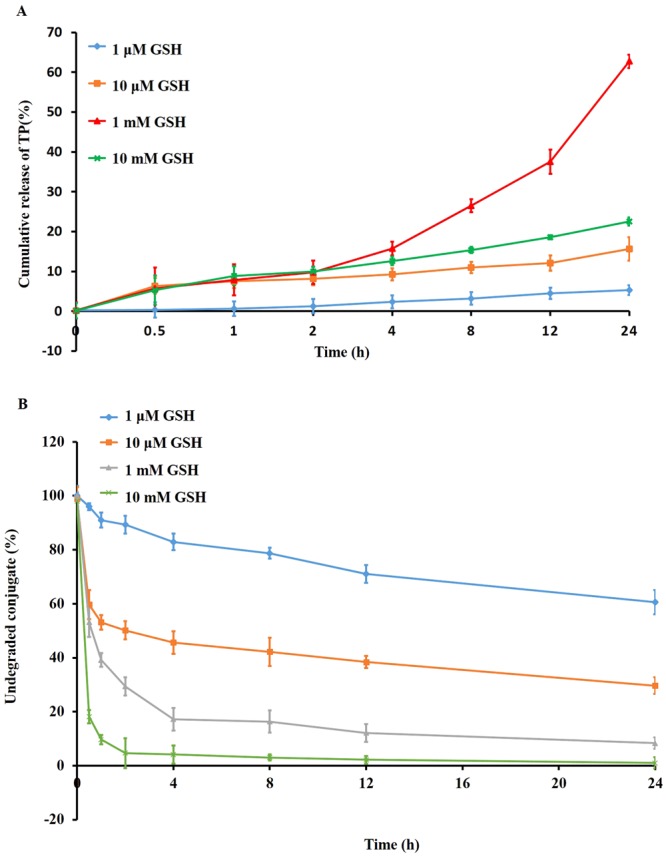
*In vitro* release studies. The GSH-sensitive release of TP (**A**) and degradation of TP prodrug (**B**) from PTPPSN studied at 37 °C under four different conditions, i.e, 10 m M, 1 m M, 10 μM, and 1 μM GSH, respectively (n = 6).

Cumulative drug release from PTPPSN, TPPSN and TP were studied in different concentrations of GSH (1 μM, 10 μM, 1 mM, and 10 mM) at pH 7.4. As shown in Fig. 5A, the cumulative release rate of PTPPSN was released fastest in 1 mM of GSH at 24 h; The cumulative release of TP was 5.29%, 15.63%, 62.75% and 22.54% at 24 h in different concentration of GSH (1 μM, 10 μM, 1 mM, and 10 mM), respectively. The degradation rate of PTPPSN at 24 h was 60.56%, 29.66%, 8.39%, and 1.01%, respectively (Fig. 5B). The release study at different pH level (6.8 and 7.4) were shown in S1.

### *In vitro* efficacy studies

**Figure 6:**
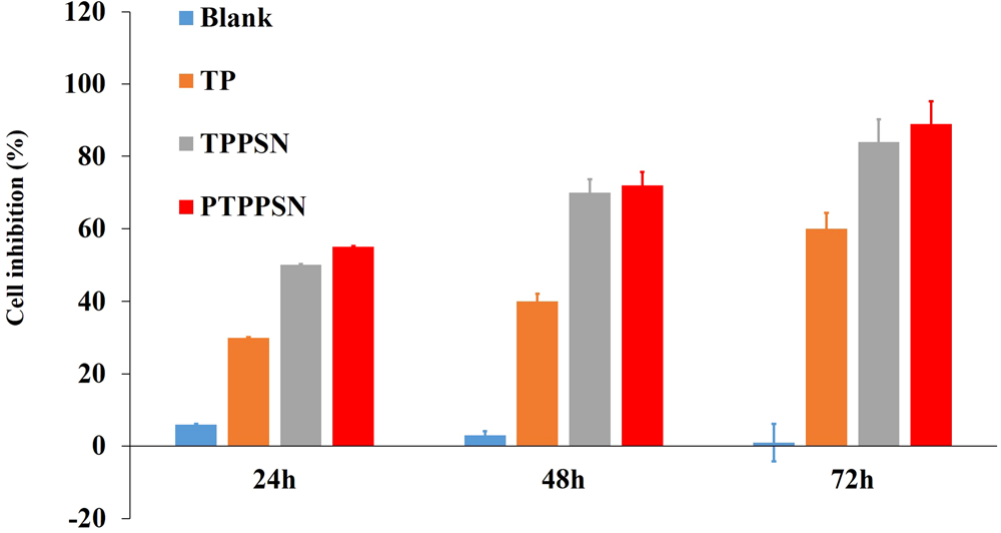
*In vitro* antitumor effect of PTPPSN.

The *in vitro* cytotoxic activity of PTPPSN against human cancer lines MCF-7 was evaluated in comparison with TP, TP prodrug, and controlled PBS. The cell viability was also verified using MTT assay. The results, presented in Fig. 6, show the concentrations required to inhibit cell growth by 50% (IC50 values). Thus, the antitumor efficacy of the self-assembled nanoparticle constructed with this prodrug has been further investigated in mice bearing an H_22_ solid tumor.

### *In vivo* antitumor efficacy

**Figure 7:**
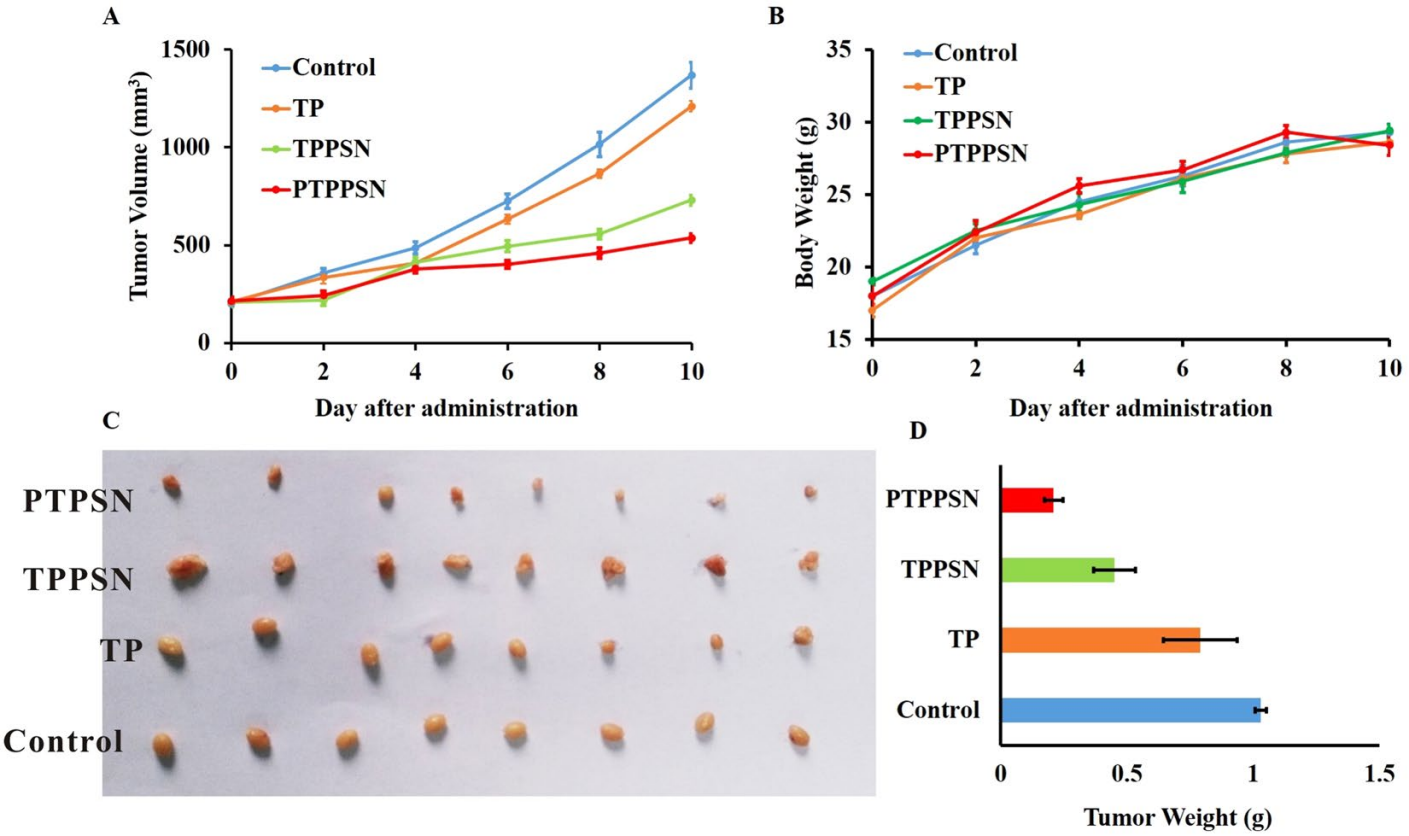
Graph showing tumor volume of solid tumor mice in different treatment groups. (**A**) antitumor effect in terms of tumor growth (error bars are mean ± SD, n = 10); (**B**) the change of body weight during the treatments; (**C**) tumor growth after systemic application of different treatment groups; (**D**) tumor weight (error bars are mean ± SD, n = 10).

The antitumor efficacy of the PTPPSN was investigated on the mice H_22_ solid tumor-burdened model, in comparison with TP, TP prodrug, and TPPSN. After tumors had reached approximately 1 cm^3^, the animals were subjected to different treatments using injection protocols, as explained in Section 2.8. As indicated in Fig. 7, the treatment with TP prodrug, TPPSN, and PTPPSN reduced the tumor volume by 11.68%, 46.72%, and 60.88%, respectively, at day 30 (p < 0.01). However, no absolute weight loss was observed in all the treated groups.

### Tissue distribution

**Figure 8:**
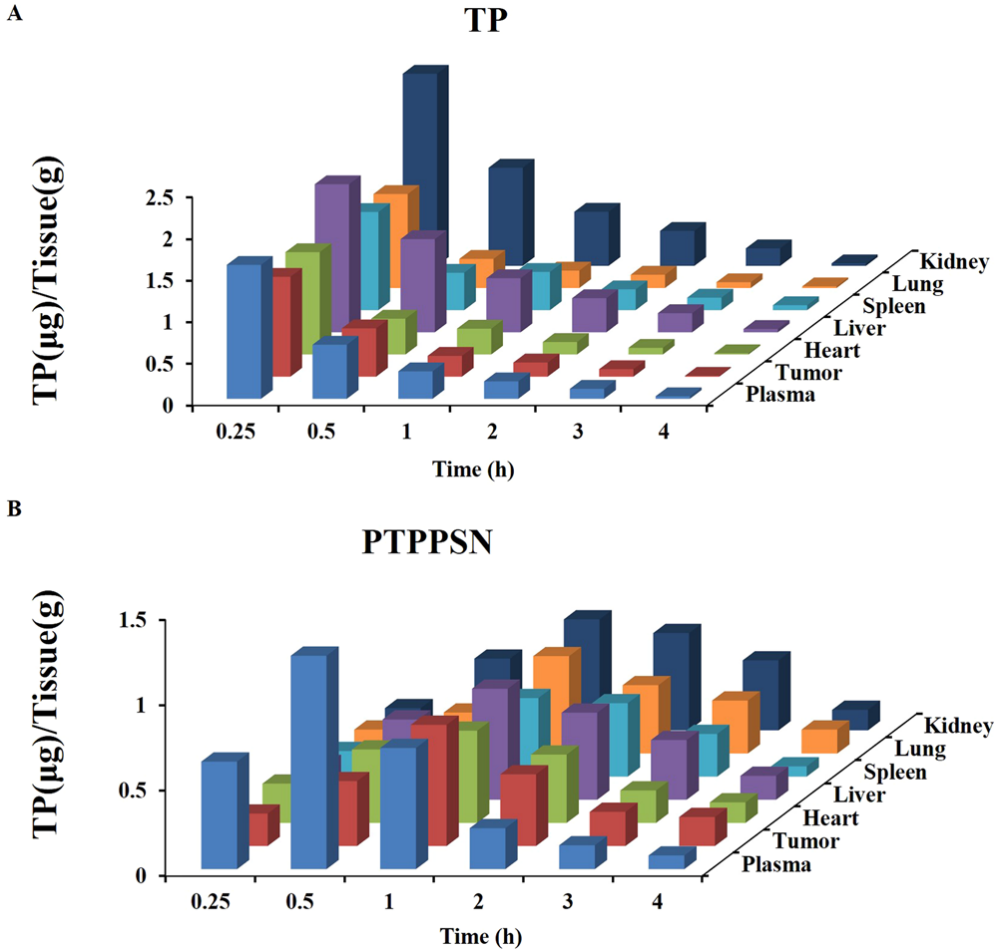
Tissue distribution of PPM after intravenous administration of TP and PTPPSN injection in solid tumor mice in blood, tumor, liver, spleen, lung, kidney and heart.

TP and PTPPSN organ distribution results were shown in Fig. 8, the kinetic study of nanoparticles was shown in S2, and the HPLC spectra of TP with retention time can be found in S3. After PEGylating TP prodrug self-assembly nanoparticles (PTPPSN) preparation, TP distributed mainly in the liver, spleen and lung, especially the liver. After 1 h of administration, the distribution of PTPPSN was significantly higher in the mice tumor than TP. This was consistent with previous reports which suggested that nanoparticles are easily identified by mononuclear phagocytic system and are enriched in mononuclear macrophages of liver, spleen and lung^38^, and the

## Discussion

Triptolide is pharmacologically potent, but its diverse toxic effects on organs such as liver, kidney, skin, gastrointestinal tract as well as the cardiac and reproductive system have restricted its therapeutic window^39^. Herein, we tried to reduce the toxicity of triptolide while enhancing its anti-tumor suppression activity. The PTPPSN was found to be a highly efficient low toxic tumor therapy hence could solved the systemic toxicity of triptolide. This could be due to PEGylating of the prodrug which prolonged release in plasma and Redox-responsive targeted at the tumor part.

With the development of nanotechnology, more nanomaterials are employed in drug formulations and delivery, viz., polymer micelle, liposomes, dendritic macromolecules, microemulsion, nano gold and other metal nanoparticles^40^. Nanomaterials can play both active and passive roles to target tumor cells. Due to the wide gap between the walls of the tumor tissue and incomplete lymphatic flow, nanoparticles of 10–200 nm can be passively gathered in the microenvironment of tumor tissue. This phenomenon is known as the enhanced permeability and retention (EPR) effect. However, due to different EPR effects on various tissues, the passive targeting strategy of nanomaterials usually fails to achieve the desired target result. Active targeting refers to nanoparticles modification or connection with monoclonal antibody marked tumor cells or peptides, which can combine with the tumor cell surface receptors or specific antibodies. This study, therefore, coupled VE alongside triptolide and PEGylated to form a self-assembly of nanoparticles. The morphological observations of PTPPSN showed a uniform dispersion of spherical objects, with the size regularity being consistent with the results of particle size distribution (Fig. 3). By increasing the concentrations of mPEG2000-LD, PTPPSN became significantly smaller, while its stability also increased remarkably (Table 1).

The cumulation rate of PTPPSN in low GSH concentration (1 μM) was lower than that of high concentration (10 mM). The *in vivo* environment of plasma GSH concentration was between 1 μM–10 mM, while the concentration of GSH in tumor environment ranged between 1 mM–10 mM (Fig. 5). Hence, the slow release of PTPPSN within the plasma environment enriched the tumor tissues by EPR effect. The enriched PTPPSN in tumor was quickly released by Redox reaction. This indicated that PTPPSN owned a certain tumor targeting property. The solid tumor volume of TP, TPPSN and PTPPSN groups were significantly lower than the blank control group. Meanwhile, the inhibition rate of the solid tumor of PTPPSN was significantly higher than that of TP and TPPSN groups (Fig. 7). The results of tumor biopsy showed that TP, TP prodrug and PTPPSN could significantly inhibit tumor growth. The PTPPSN and TP were mainly distributed in organs like liver, spleen and lung but maximally in the liver (Fig. 8). The distribution results suggested that PTPPSN can effectively circumvent the mononuclear scavenger system identification through the cumulative EPR effect on tumor site.

In conclusion, this study demonstrated that more efficient diterpene lactones based anticancer nanomedicines could be designed by conjugating with VE, thereby potentiating their “target recognition” of the tumor microenvironment.

## Electronic supplementary material

Supplementary Information
