# Supplementary material for: Redox-responsive self-assembly PEG nanoparticle enhanced triptolide for efficient antitumor treatment
Source: Sci Rep. 2018 Aug 28;8:12968. doi: 10.1038/s41598-018-29692-0 (PMC6113257; doi:10.1038/s41598-018-29692-0)
Supplement: Supplementary file 1 — Supplementary Information [file 41598_2018_29692_MOESM1_ESM.pdf]

Supplementary

Redox-responsive self-assembly PEG nanoparticle enhanced triptolide for efficient antitumor treatment

Yanchun Wang[1, 2, $], Xuewei Liu[3, $], Xuemei Wang[1, 2], Wei Zheng[4], Junping Zhang[4] , Feng Shi[5], Junbao Liu[1, 3, *]

1, People's Hospital of Zhengzhou University, Zhengzhou, 450003, Henan, P.R. China

2, Henan Provincial People's Hospital, Zhengzhou, 450003, Henan, P.R. China

3, Henan University of Traditional Chinese Medicine, Zhengzhou, Henan, P.R. China,

4, Henan Academy Institute of Traditional Chinese Medicine 45000, Zhengzhou, P.R. China

5, Jiangsu University, 212013, Zhenjiang, P.R. China

$, co-authorship

*, corresponding author's: ycwang450003@gmail.com

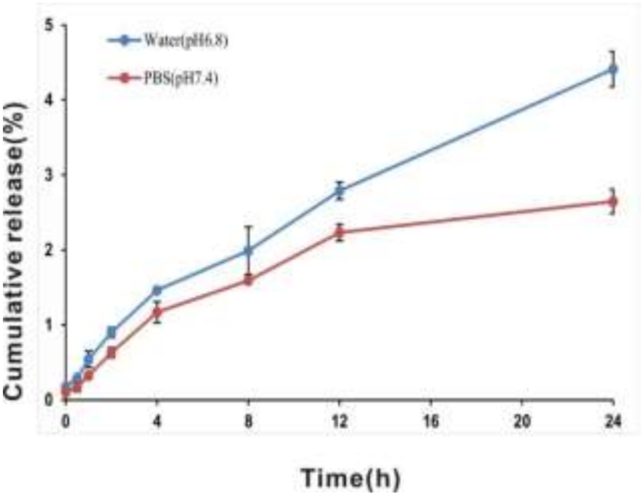

S1 In vitro release studies of PTPPSN studied in at 37 °C under different pH (n = 6).

S 2 Plasma pharmacokinetic parameters of intravenously injected PTPPSN in mice

	AUC 0-240 (μg/ml·min)	C_{MAX} (μg/ml)	$T_{1/2}$ (min)	MRT_{0-t} (min)	T_{MAX} (min)
Free TP	4.17 ± 0.058	0.031 ± 0.0017	103.61 ± 3.77	83.21 ± 2.17	15
PTPPSN	90.23 ± 0.089	1.25 ± 0.058	44.812 ± 1.18	68.72 ± 0.99	30

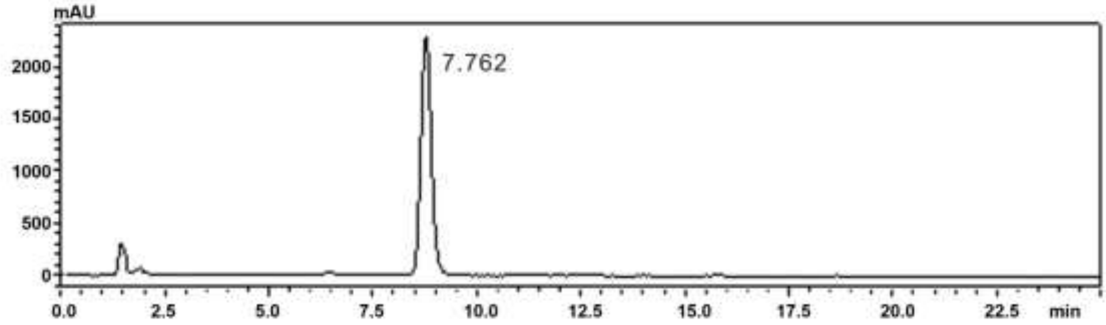

S3 The HPLC spectra of the Triptolide
